# Nanostructure, structural stability, and diffusion characteristics of layered coatings for heat-assisted magnetic recording head media

**DOI:** 10.1038/s41598-018-27688-4

**Published:** 2018-06-28

**Authors:** J. Matlak, E. Rismaniyazdi, K. Komvopoulos

**Affiliations:** 1 Department of Mechanical Engineering, University of California, Berkeley, CA 94720 USA; 2 Western Digital Company, San Jose, CA 95119 USA

## Abstract

The intense laser heating in heat-assisted magnetic recording (HAMR) has been a major hindrance to HAMR technology from becoming commercially viable. Thermal damage of the near-field transducer (NFT) and write pole (WP) embedded in the trailing edge of the magnetic head due to failure of the protective carbon overcoat after prolonged heating at an elevated temperature are major obstacles. Therefore, the main objective of this study was to develop an effective coating method for HAMR heads. This was accomplished by introducing a new class of layered coatings consisting of ultrathin amorphous carbon (*a*-C) overcoat, adhesion (SiN) layer, and buffer (NiCr or TaO_x_) layer sequentially deposited onto Au and FeCo base layers to mimic the layer stacking of NFT and WP elements, respectively. The structural stability of the *a*-C overcoats and diffusion characteristics of each comprising layer under conditions of heating at 350 °C for 30 min in an Ar atmosphere were investigated by high-resolution transmission electron microscopy (HRTEM), scanning transmission electron microscopy (STEM), and electron energy loss spectroscopy (EELS). For most stacking configurations the HRTEM/STEM and EELS results generally revealed some layer intermixing and minute carbon atom rehybridization in the heated *a*-C overcoats. The findings of this investigation suggest that further optimization of the developed layered coatings can provide a viable solution to thermal damage of HAMR heads.

## Introduction

Amorphous carbon (*a*-C) films are commonly used in commercial hard-disk drives as overcoats of both magnetic heads and hard disks to provide protection against corrosion and mitigate surface damage from intermittent impact events. A main reason for the remarkable increase in storage capacity over the past two decades has been the development of much thinner overcoats and the significant decrease of the head-disk spacing. Industry attempts to transition to a new storage technology, namely heat-assisted magnetic recording (HAMR), which promises to further increase the areal density to levels of 1–5 Tb/in^2^ and beyond^1–3^, have focused the attention on the overcoat’s long-term capability to protect vital head components subjected to intense thermal conditions^4–6^. Reliability issues^7^ have been proven an immense challenge, however, delaying commercialization of HAMR technology, or even resulting in prioritizing alternative technologies, such as microwave-assisted magnetic recording^8^. Resolving such critical issues and developing robust protective overcoats that can withstand continuous exposure to elevated temperatures without loss of their protective capability is therefore critical to the successful implementation of HAMR technology.

In a typical HAMR hard-disk drive, a laser-optical system is integrated with the magnetic head, and a waveguide coupled with a near-field transducer (NFT) focusses the laser beam to a tiny surface spot. The resulting intense localized heating of the magnetic medium above its Curie temperature^1^ lowers the medium’s coercivity significantly, enabling data to be written by the write pole (WP) in very tightly packed magnetic domains to increase the areal density. An ultrathin carbon overcoat is deposited onto the NFT and WP for protection against oxidation and mechanical wear. Additional layers, such as a SiN adhesion (seed) layer and a NiCr or TaO_x_ diffusion (buffer) layer^9^, may also be deposited beneath the overcoat to help curb oxidation and diffusion of elements and also facilitate heat transfer, especially at elevated temperatures^10,11^.

The absorption and dissipation of laser energy in the vicinities of the NFT and WP raises locally the temperature by 100–300 °C, which destabilizes the overcoat through oxidation and graphitization processes, promotes interdiffusion, and accelerates the deterioration of NFT and WP elements^12^. The degradation of the overcoat over the WP is of particular concern because the iron alloy that it is made of is more sensitive to oxidation than the gold NFT. Carbon films may disintegrate (oxidize) at temperatures as low as 300 °C in an atmospheric environment^13,14^, which is within the steady-state operating temperature range of a HAMR head. Both the mechanical performance^15^ and thermal stability of the carbon overcoat depends on the content of tetrahedral (*sp*^3^) and trigonal (*sp*^2^) carbon atom hybridization. Highly tetrahedral (>80% s*p*^3^) *a*-C films are thermally stable up to ~1000 °C, exhibiting negligible graphitization and minimal carbon bonding rehybridization, whereas *a*-C films with 40% s*p*^3^ graphitize at ~400 °C^16^. In most contemporary hard-disk drives, the carbon overcoat thickness is ≲5 nm; therefore, the lower threshold of minimum s*p*^3^ content is encouraging given the difficulty to synthesize ultrathin films with a sufficiently thick *sp*^3^-rich bulk layer^17^.

Select deposition systems, particularly plasma-enhanced chemical vapor deposition^18,19^ and filtered cathodic vacuum arc (FCVA)^20,21^, produce continuous ultrathin carbon films exhibiting predominantly *sp*^3^ hybridization^22^. FCVA enables low-temperature deposition of *a*-C films with excellent nanomechanical properties and structural stability at an elevated temperature^16^, provided they are sufficiently thick^23^. Therefore, the FCVA method was selected in this study to deposit ultrathin *a*-C overcoats under optimum plasma conditions.

The main objective of this study was to investigate the structural stability and diffusion characteristics of layered coatings at an elevated temperature typical of the writing process and assess their potential as protective overcoats of HAMR heads. Layered coatings consisting of *a*-C overcoat, SiN adhesion layer, and NiCr or TaO_x_ buffer layer were deposited onto Au or FeCo base layers deposited onto a Si(100) substrate. The main role of the adhesion SiN layer is to enhance the bond strength of the *a*-C overcoat, whereas that of the NiCr or TaO_x_ buffer layer is to prevent oxidation and interdiffusion of carbon into the base metal layer. High-resolution transmission electron microscopy (HRTEM), scanning transmission electron microscopy (STEM), and electron energy loss spectroscopy (EELS) were used to examine changes in the *a*-C overcoat nanostructure with respect to atomic carbon bond hybridization as well as overcoat intermixing with underlying adhesion, buffer, and base layers due to heating under conditions resembling those encountered during steady-state operation of a HAMR head. The HRTEM/STEM and EELS results of various layered coating configurations, i.e., *a*-C/SiN/NiCr(TaO_x_)/Au(FeCo), are contrasted to assess the integrity and protective capability of these new class of coatings under thermal conditions typical of HAMR heads.

## Results

### NFT layered coatings

**Figure 1:**
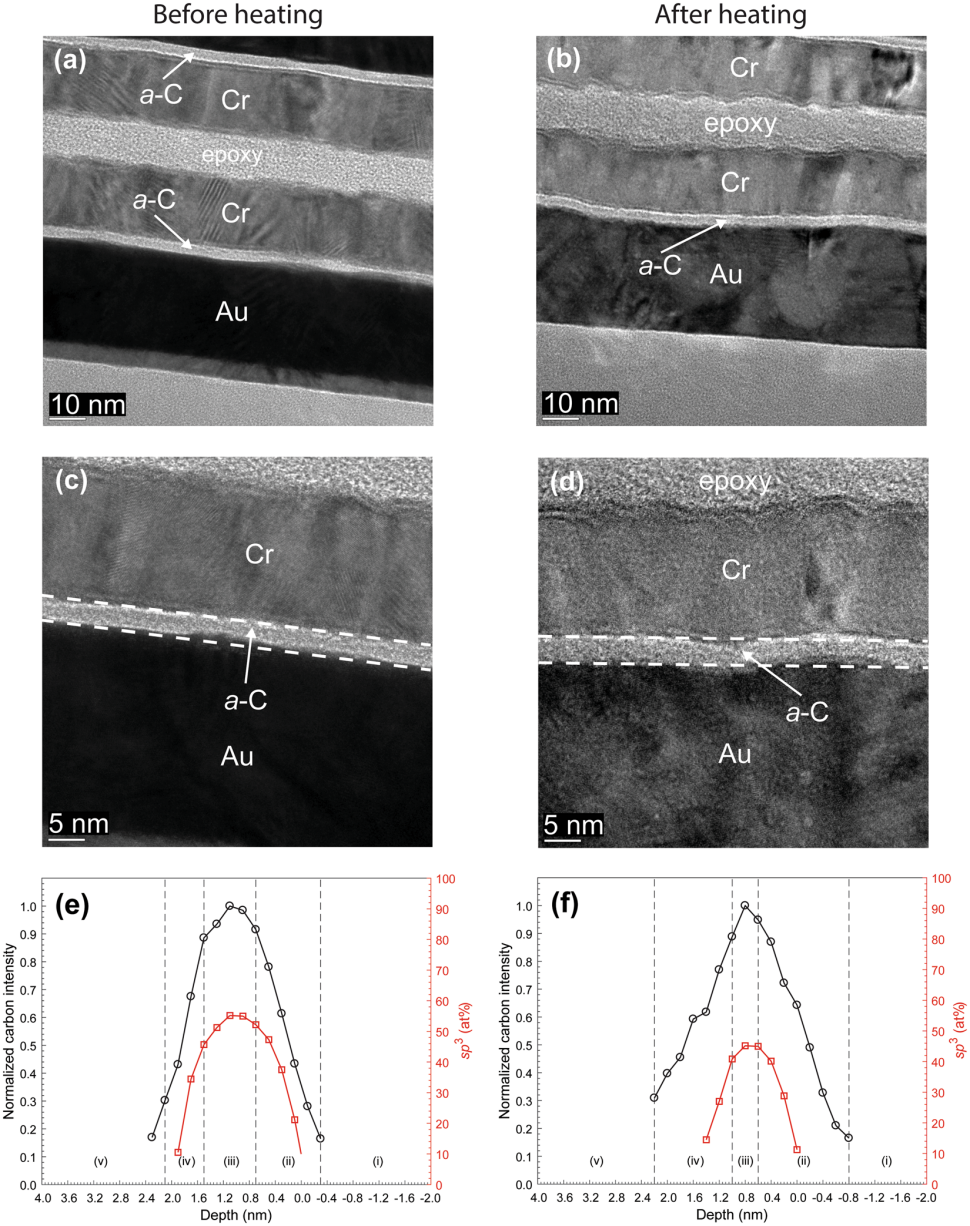
(**a**–**d**) Cross-sectional HRTEM images and (**e**,**f**) depth profiles of C K-edge normalized intensity and *sp*^3^ fraction calculated from C K-edge EELS spectra of industry sourced *a*-C overcoats deposited on a SiN/Au sample stack obtained before and after heating at 350 °C for 30 min in an Ar environment. (The EELS profiles show a layered cross-sectional nanostructure consisting of the following five regions: (i) underlayer, (ii) interface (intermixing) layer, (iii) bulk layer, (iv) surface layer, and (v) capping layer).

**Figure 2:**
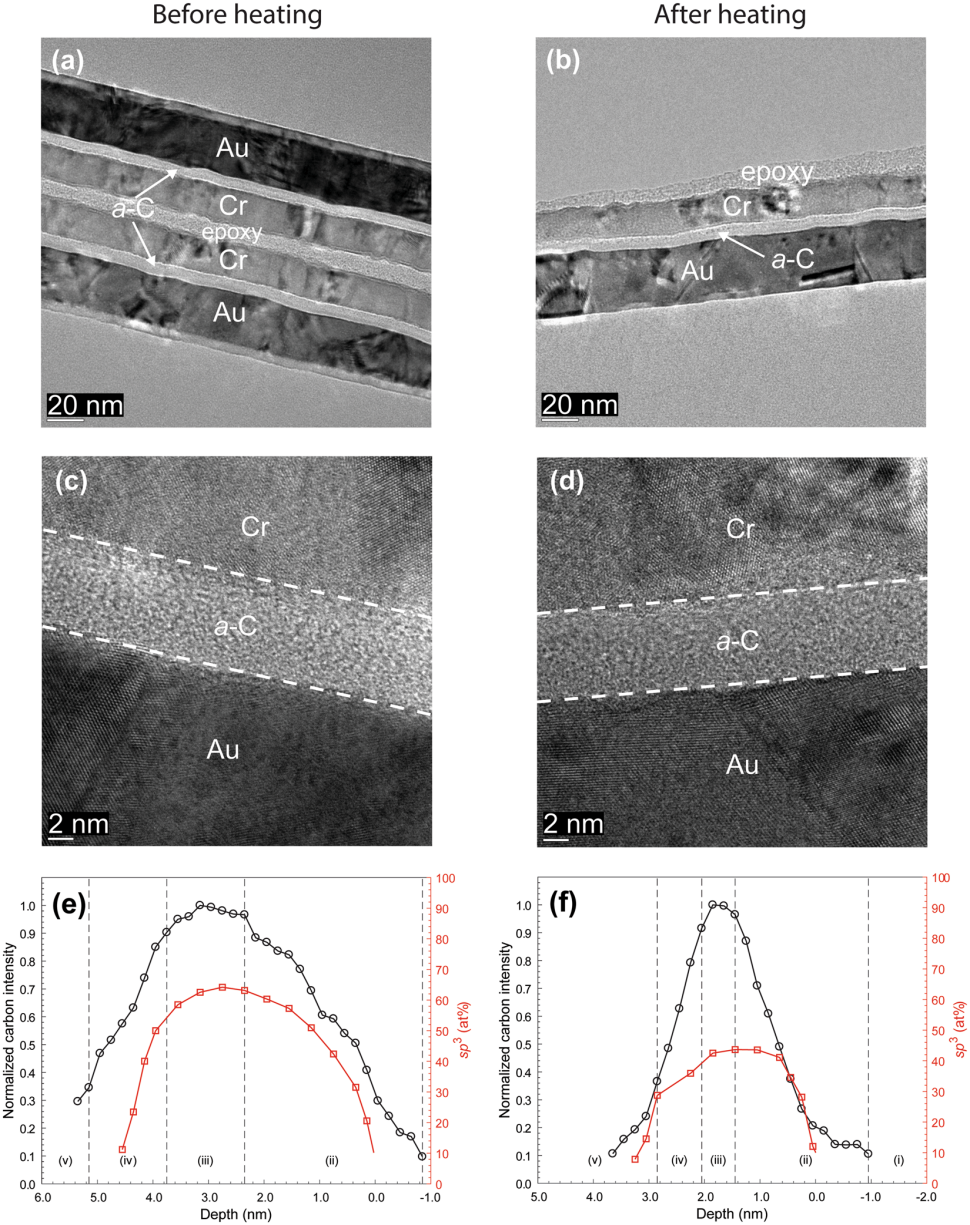
(**a**–**d**) Cross-sectional HRTEM images and (**e,f**) depth profiles of C K-edge normalized intensity and *sp*^3^ fraction calculated from C K-edge EELS spectra of *a*-C overcoats deposited on a SiN/Au sample stack obtained before and after heating at 350 °C for 30 min in an Ar environment. (The EELS profiles show a layered cross-sectional nanostructure consisting of the following five regions: (i) underlayer, (ii) interface (intermixing) layer, (iii) bulk layer, (iv) surface layer, and (v) capping layer).

**Figure 3:**
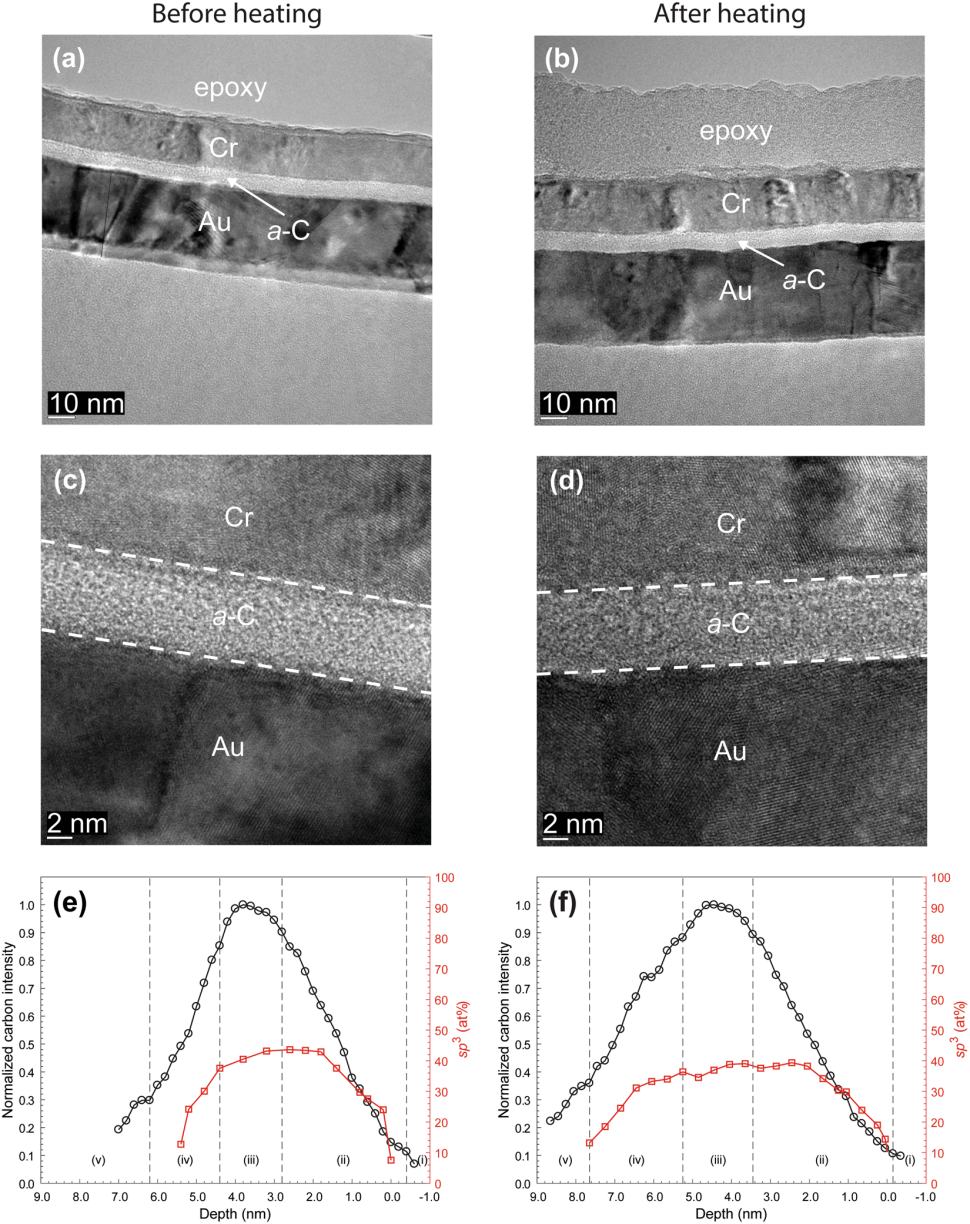
(**a**–**d**) Cross-sectional HRTEM images and (**e**,**f**) depth profiles of C K-edge normalized intensity and *sp*^3^ fraction calculated from C K-edge EELS spectra of *a*-C overcoats deposited on a SiN/NiCr/Au sample stack obtained before and after heating at 350 °C for 30 min in an Ar environment. (The EELS profiles show a layered cross-sectional nanostructure consisting of the following five regions: (i) underlayer, (ii) interface (intermixing) layer, (iii) bulk layer, (iv) surface layer, and (v) capping layer).

Figures 1–3 show HRTEM images and EELS depth profiles of normalized carbon intensity and *sp*^3^ fraction of *a*-C/SiN/Au and *a*-C/NiCr/Au sample stacks before and after heating. The results of industry sourced *a*-C overcoats (Fig. 1) are contrasted with those of *a*-C overcoats developed in this study (Figs 2 and 3). The HRTEM images show the order of sample stack and provide insight into the overcoat’s uniformity and approximate thickness, while the EELS plots reveal the overcoat’s cross-sectional nanostructure and composition. Structure and contrast differences in the HRTEM images reveal the presence of the *a*-C overcoat and Au base layer; however, it is difficult to distinguish the SiN and NiCr layers based on contrast differences alone.

A comparison of the HRTEM images of each sample stack before and after heating shows minimal changes in thickness and continuity of each layer, with only slight layer deformation, which is most likely due to the thermal expansion coefficient mismatch between the overcoat and underlying layer. The nominal thickness of *a*-C overcoats (Table 1), measured from high-magnification HRTEM images, is equal to 3 nm for industry sourced *a*-C overcoats (Fig. 1c,d) and 6-7 nm for custom-made *a*-C overcoats (Figs 2c,d and 3c,d).

**Table 1:** Layer composition and nominal thickness in investigated stacking configurations.

Base layer	Layer composition				Nominal layer thickness^[e]^ (nm)			
	Layer 1	Layer 2	Layer 3	Layer 4	Layer 1	Layer 2	Layer 3	Layer 4
Au	—	—	SiN^[a]^	*a*-C^[c]^	—	—	0.8	3.1 ± 0.3
	—	—	SiN^[b]^	*a*-C	—	—	3.0	7.2 ± 0.1
	NiCr	—	SiN^[b]^	*a*-C	1.0	—	3.0	6.8 ± 0.1
FeCo	—	—	SiN^[a]^	*a*-C^[c]^	—	—	0.8	3.6 ± 0.1
	—	—	SiN^[b]^	*a*-C	—	—	3.0	5.4 ± 0.1
	NiCr	—	SiN^[b]^	*a*-C	1.0	—	3.0	5.3 ± 0.1
	—	TaO_x_	SiN^[b]^	*a*-C	—	4.0	3.0	9.4 ± 0.3

^[a]^Type I.

^[b]^Type II.

^[c]^Industry sourced (synthesized by filtered cathodic vacuum arc using zero substrate bias voltage, 90° incidence angle, and ~79 s deposition time).

^[e]^The thickness of layers 1–3 is the apparent thickness, while the thickness of layer 4 was measured from the corresponding HRTEM images.

The EELS depth profiles of normalized carbon intensity and *sp*^3^ content of *a*-C overcoats deposited on SiN/Au (Figs 1e,f and 2e,f) and SiN/NiCr/Au (Fig. 3e,f) sample stacks before and after heating show a layered cross-sectional nanostructure consisting of the following five regions: (i) underlayer, (ii) interface (intermixing) layer, (iii) bulk layer (rich in *sp*^3^ hybridization), (iv) surface layer, and (v) capping layer. The interfaces of these regions are defined in Table 2. The underlying physical mechanisms responsible for the growth of *a*-C overcoats with such multilayer cross-sectional structures have been discussed elsewhere^17^. A comparison of the depth profiles of each sample reveals a consistent decrease in *sp*^3^ content after heating, which ranges between 4 and 16% (Table 3). This is most likely due to partial *sp*^3^→*sp*^2^ rehybridization of the overcoats, as carbon films tend to destabilize at elevated temperatures^16^.

**Table 2:** Interfaces in layered cross-sectional nanostructure of *a*-C overcoats determined from the normalized C-K edge intensity and the *sp*^3^ content.

Layer interface	*sp*^3^/*sp*^2^ transition	C-K edge normalized intensity range
Base layer/intermixing layer	From approximately zero to rapidly increasing	<0.15
Intermixing layer/bulk layer	From rapidly increasing to approximately constant value	0.9–1.0
Bulk layer/surface layer	From approximately constant value to rapidly decreasing	0.85–0.95
Capping layer/surface layer	From decreasing to low stable value due to adventitious carbon	0.3–0.4

**Table 3:** Maximum *sp*^3^ content of bulk layer (calculated from EELS measurements) of the *a*-C overcoat in investigated stacking configurations before and after heating.

Base layer	Layer composition				Maximum *sp*^3^ content of bulk layer (at%)		
	Layer 1	Layer 2	Layer 3	Layer 4	Before heating	After heating	Change (%)
Au	—	—	SiN^[a]^	*a*-C^[c]^	55	45	−10
	—	—	SiN^[b]^	*a*-C	64	48	−16
	NiCr	—	SiN^[b]^	*a*-C	44	40	−4
FeCo	—	—	SiN^[a]^	*a*-C^[c]^	53	49	−4
	—	—	SiN^[b]^	*a*-C	56	49	−7
	NiCr	—	SiN^[b]^	*a*-C	31	23	−8
	—	TaO_x_	SiN^[b]^	*a*-C	55	49	−6

^[a]^Type I.

^[b]^Type II.

^[c]^Industry sourced (synthesized by filtered cathodic vacuum arc using zero substrate bias voltage, 90° incidence angle, and ~79 s deposition time).

### WP layered coatings

**Figure 4:**
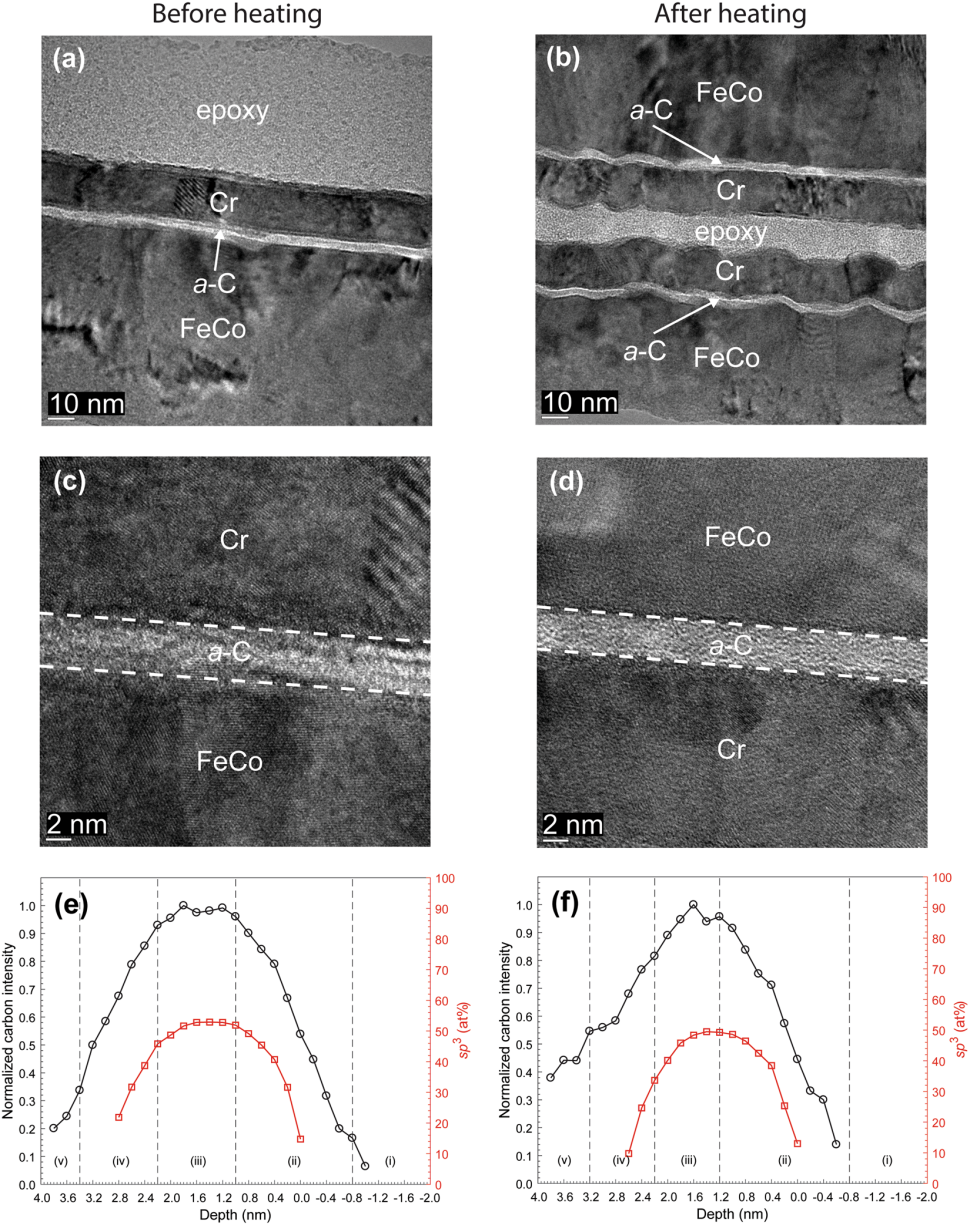
(**a**–**d**) Cross-sectional HRTEM images and (**e**,**f**) depth profiles of C K-edge normalized intensity and *sp*^3^ fraction calculated from C K-edge EELS spectra of industry sourced *a*-C overcoats deposited on a SiN/FeCo sample stack obtained before and after heating at 350 °C for 30 min in an Ar environment. (The EELS profiles show a layered cross-sectional nanostructure consisting of the following five regions: (i) underlayer, (ii) interface (intermixing) layer, (iii) bulk layer, (iv) surface layer, and (v) capping layer).

**Figure 5:**
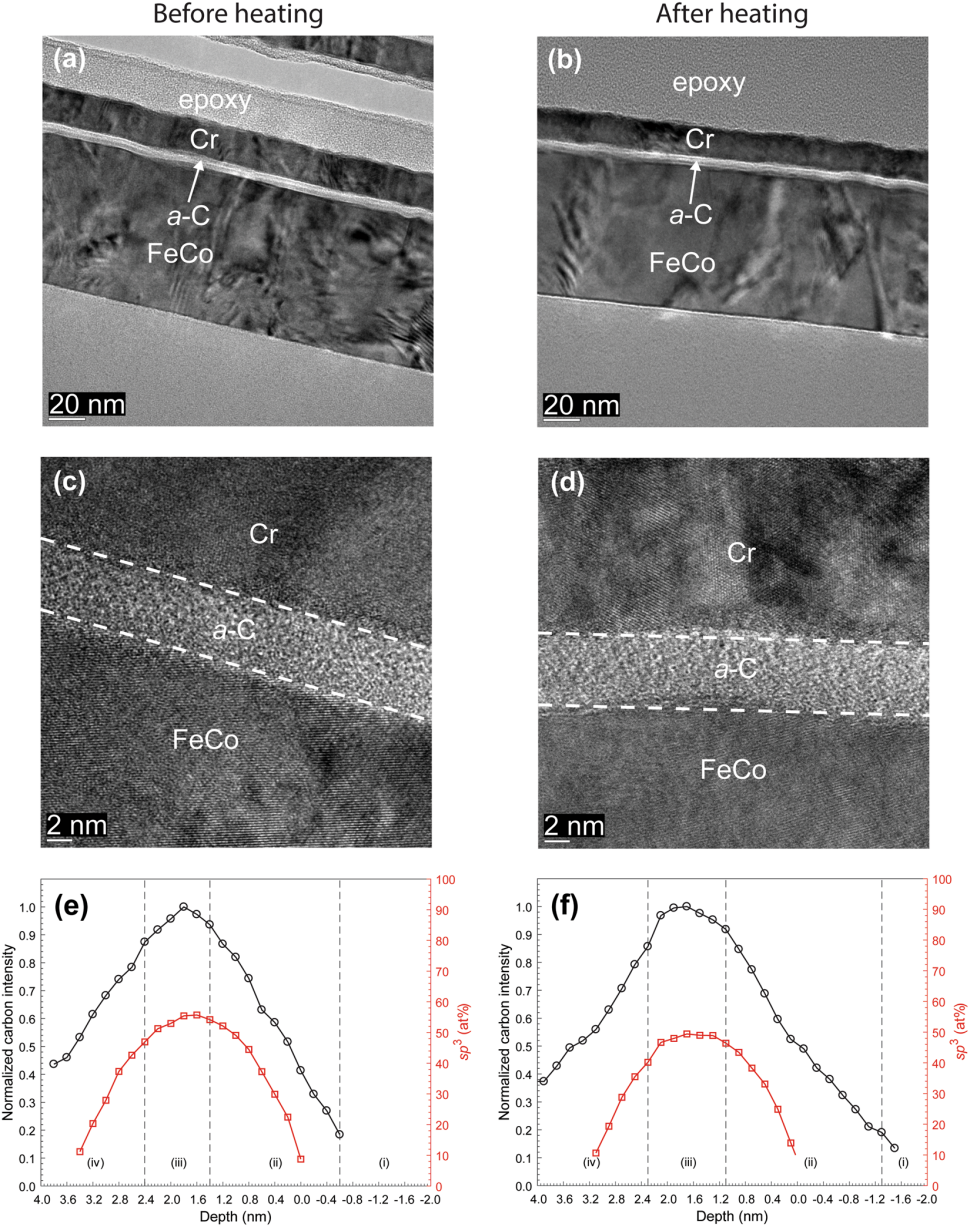
(**a**–**d**) Cross-sectional HRTEM images and (**e**,**f**) depth profiles of C K-edge normalized intensity and *sp*^3^ fraction calculated from C K-edge EELS spectra of *a*-C overcoats deposited on a SiN/FeCo sample stack obtained before and after heating at 350 °C for 30 min in an Ar environment. (The EELS profiles show a layered cross-sectional nanostructure consisting of the following five regions: (i) underlayer, (ii) interface (intermixing) layer, (iii) bulk layer, (iv) surface layer, and (v) capping layer).

**Figure 6:**
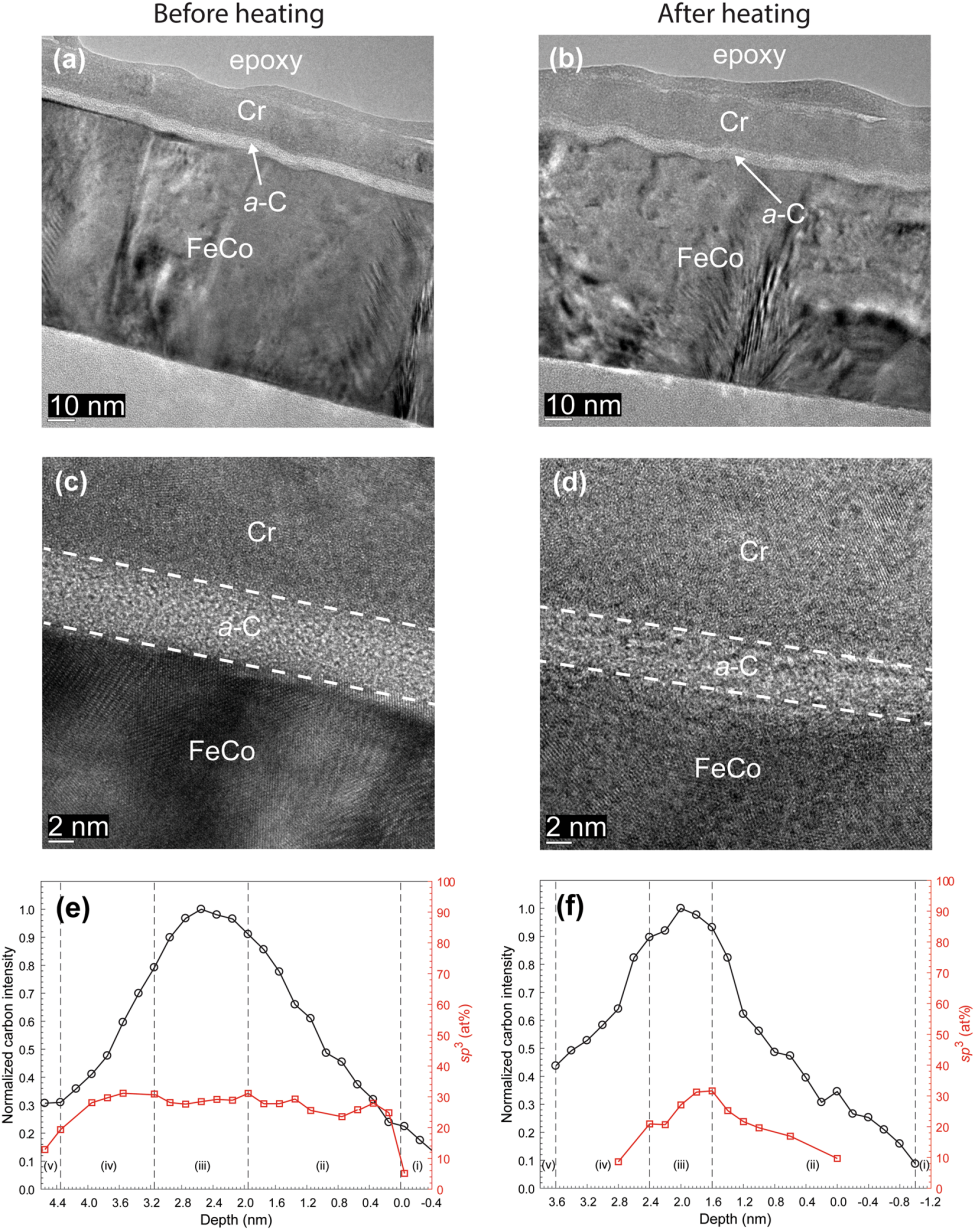
(**a**–**d**) Cross-sectional HRTEM images and (**e**,**f**) depth profiles of C K-edge normalized intensity and *sp*^3^ fraction calculated from C K-edge EELS spectra of *a*-C overcoats deposited on a SiN/NiCr/FeCo sample stack obtained before and after heating at 350 °C for 30 min in an Ar environment. (The EELS profiles show a layered cross-sectional nanostructure consisting of the following five regions: (i) underlayer, (ii) interface (intermixing) layer, (iii) bulk layer, (iv) surface layer, and (v) capping layer).

**Figure 7:**
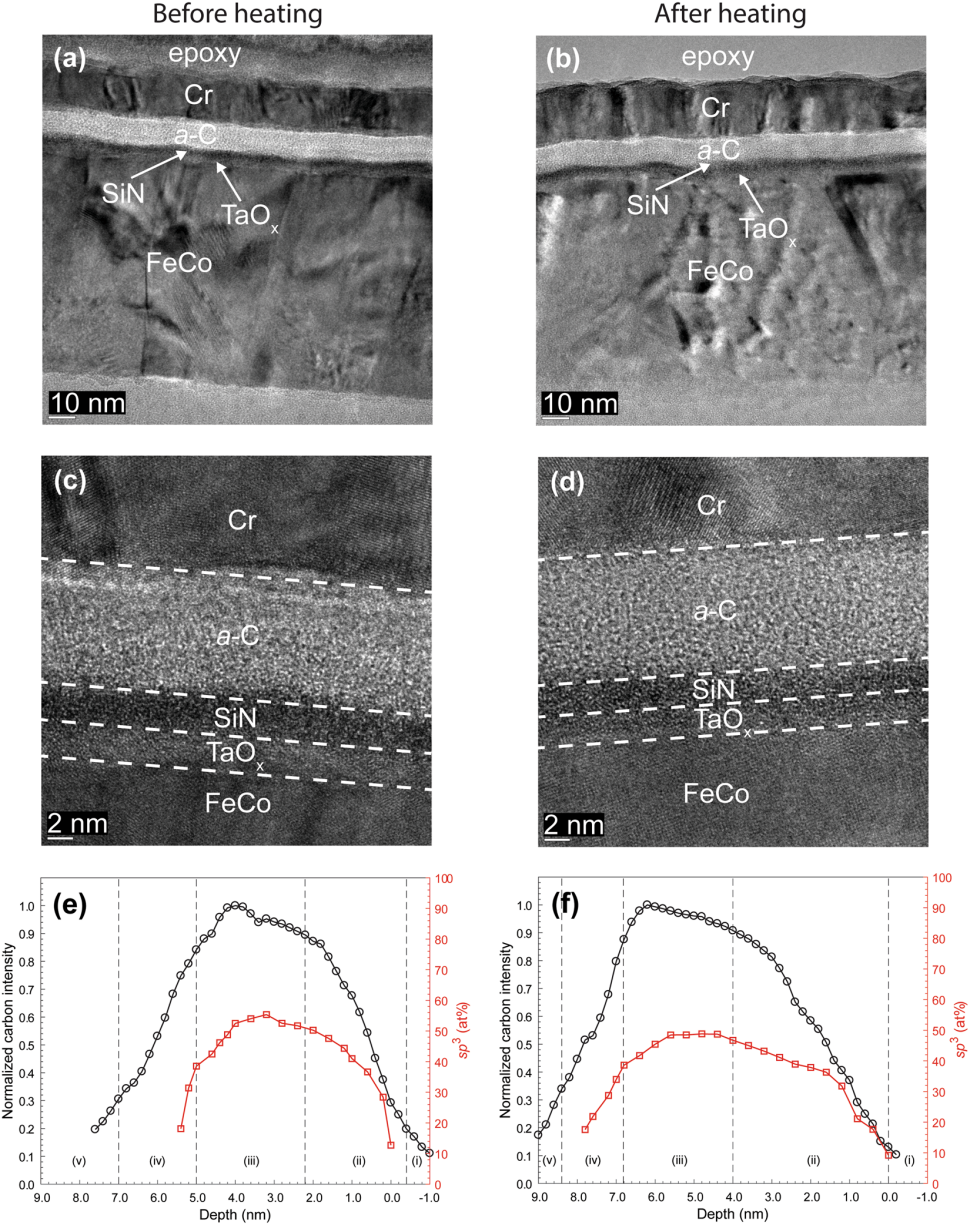
(**a**–**d**) Cross-sectional HRTEM images and (**e**,**f**) depth profiles of C K-edge normalized intensity and *sp*^3^ fraction calculated from C K-edge EELS spectra of *a*-C overcoats deposited on a SiN/TaO_x_/FeCo sample stack obtained before and after heating at 350 °C for 30 min in an Ar environment. (The EELS profiles show a layered cross-sectional nanostructure consisting of the following five regions: (i) underlayer, (ii) interface (intermixing) layer, (iii) bulk layer, (iv) surface layer, and (v) capping layer).

Figures 4–7 show HRTEM images and EELS depth profiles of normalized carbon intensity and *sp*^3^ fraction of *a*-C/SiN/FeCo, *a*-C/NiCr/FeCo, and *a*-C/TaO_x_/FeCo sample stacks before and after heating. Results of industry sourced *a*-C overcoats (Fig. 4) are again compared with results of *a*-C overcoats developed in this study (Figs 5–7). As before, the HRTEM images show the order of sample stack and the overcoat’s uniformity and approximate thickness, whereas the EELS plots reveal the overcoat’s cross-sectional nanostructure and composition. Contrast and structure differences in the HRTEM images show the presence of *a-*C overcoat, TaO_x_ buffer layer, and FeCo base layer; however, it is not possible to distinguish the SiN and NiCr layers based on contrast differences alone.

Similar to samples with an Au base layer, a comparison of the HRTEM images of each sample stack before and after heating shows minimal changes in thickness and continuity of each layer. The nominal overcoat thickness (Table 1), measured from high-magnification HRTEM images, is equal to 3 nm for industry sourced *a*-C overcoats (Fig. 4c,d) and 5 or 9 nm for custom-made *a*-C overcoats (Figs 5c,d; 6c,d; and 7c,d).

The EELS depth profiles of normalized carbon intensity and *sp*^3^ content of *a*-C overcoats deposited onto SiN/FeCo (Figs 4e,f and 5e,f), SiN/NiCr/FeCo (Fig. 6e,f), and SiN/TaO_x_/FeCo (Fig. 7e,f) sample stacks before and after heating show a layered cross-sectional nanostructure consisting of same five regions as those observed with sample stacks having an Au base layer and interfaces defined in Table 2. A comparison of the depth profiles of each sample stack shows a consistent but minute decrease in overcoat *sp*^3^ content due to heating ranging between 4 and 8% (Table 3), which is again attributed to partial *sp*^3^→*sp*^2^ rehybridization of the *a*-C overcoats.

### Carbon overcoat thickness before and after heating

Table 4 gives the thickness of each layer in different stack configurations determined from EELS measurements before and after heating. The calculation of the thickness data was based on the extents along a scan line where the elemental peaks of a characteristic layer material exist. Unlike standard techniques of estimating or measuring the film thickness, EELS provides information about the true thickness, including the film portion intermixed with other layers. As a result, the layer thickness calculated from EELS measurements may be larger than what might be inferred from HRTEM images, where it is much more difficult to distinguish the intermixing regions, or even the nominal layer thickness in the case of indistinguishable contrast differences. Thus, the larger values of layer thickness deduced from the EELS measurements can be attributed to the inclusion of the intermixing regions in the calculated layer thickness, which extend beyond the pure elemental layers seen in the HRTEM images.

**Table 4:** Layer composition and thickness (calculated from EELS measurements) in investigated stacking configurations before and after heating.

Base layer	Layer composition				Layer thickness (nm)							
	Layer 1	Layer 2	Layer 3	Layer 4	Before heating				After heating			
					Layer 1	Layer 2	Layer 3	Layer 4	Layer 1	Layer 2	Layer 3	Layer 4
Au	—	—	SiN^[a]^	*a*-C^[c]^	—	—	—	6.3 ± 0.7	—	—	—	7.1 ± 1.3
	—	—	SiN^[b]^	*a*-C	—	—	4.9 ± 0.5	5.7 ± 0.3	—	—	4.9 ± 0.6	7.3 ± 0.5
	NiCr	—	SiN^[b]^	*a*-C	2.5 ± 0.9	—	4.7 ± 1.1	7.3 ± 0.6	3.1 ± 0.5	—	4.8 ± 0.6	8.2 ± 0.6
FeCo	—	—	SiN^[a]^	*a*-C^[c]^	—	—	—	7.9 ± 0.7	—	—	—	7.5 ± 0.8
	—	—	SiN^[b]^	*a*-C	—	—	5.8 ± 0.6	8.0 ± 0.6	—	—	5.3 ± 0.3	7.7 ± 0.4
	NiCr	—	SiN^[b]^	*a*-C	4.0 ± 0.8	—	4.8 ± 0.5	8.8 ± 1.3	4.4 ± 0.4	—	6.0 ± 0.9	6.6 ± 0.6
	—	TaO_x_	SiN^[b]^	*a*-C	—	5.0 ± 0.8	4.8 ± 0.9	10.7 ± 1.2	—	4.4 ± 1.0	7.4 ± 0.9	12.0 ± 1.0

^[a]^Type I.

^[b]^Type II.

^[c]^Industry sourced (synthesized by filtered cathodic vacuum arc using zero substrate bias voltage, 90° incidence angle, and ~79 s deposition time).

A comparison of layer thickness measurements performed before and after heating reveals several interesting trends. For all stack samples having an Au base layer and the *a*-C/SiN/TaO_x_/FeCo stack sample, the *a*-C overcoat thickness appears to have slightly increased after heating; however, the opposite trend is observed for the *a*-C overcoat in the *a*-C/SiN/NiCr/FeCo stack configuration, while the thickness of the *a*-C overcoat in the *a*-C/SiN/FeCo stack samples was not significantly affected by heating. Table 4 also shows that changes in the thickness of the adhesion and buffer layers are within experimental scatter; the only exception being the SiN adhesion layer in the *a*-C/SiN/NiCr/FeCo and *a*-C/SiN/TaO_x_/FeCo stack configurations in which the thickness increased after heating.

### Layer intermixing due to heating

Intermixing between the overcoat, adhesion, buffer, and base layers was determined from the EELS spectra by tracking the simultaneous presence of characteristic elemental peaks in the high energy loss range. NiCr was identified by the presence of the Cr L_2,3_-major edge (575–584 eV), SiN was identified by the presence of the N K-major edge (401 eV), FeCo was identified by the presence of both the Fe L_2_- and L_3_-major edges (708–721 eV) and the Co L_2_- and L_3_-major edges (779–794 eV), Au was identified by the presence of the O_2,3_-major edge (54 eV), and TaO_x_ was identified by the presence of the O K-edge (532 eV)^24^. The EELS major edges used to identify the presence of various layers in the investigated stacking configurations are shown in the Supplementary Information. Although most of the elements have distinctive peaks that were always clearly identifiable, Au was the exception. Other low energy loss features (i.e., plasmons) overlapped with the Au O_2,3_-major edge when other elements were present in the same scanned region. As a result, the transition from the Au layer to other layers could only be approximated and was defined as the point where the last two peaks of Au flattened, forming a tangent line.

**Figure 8:**
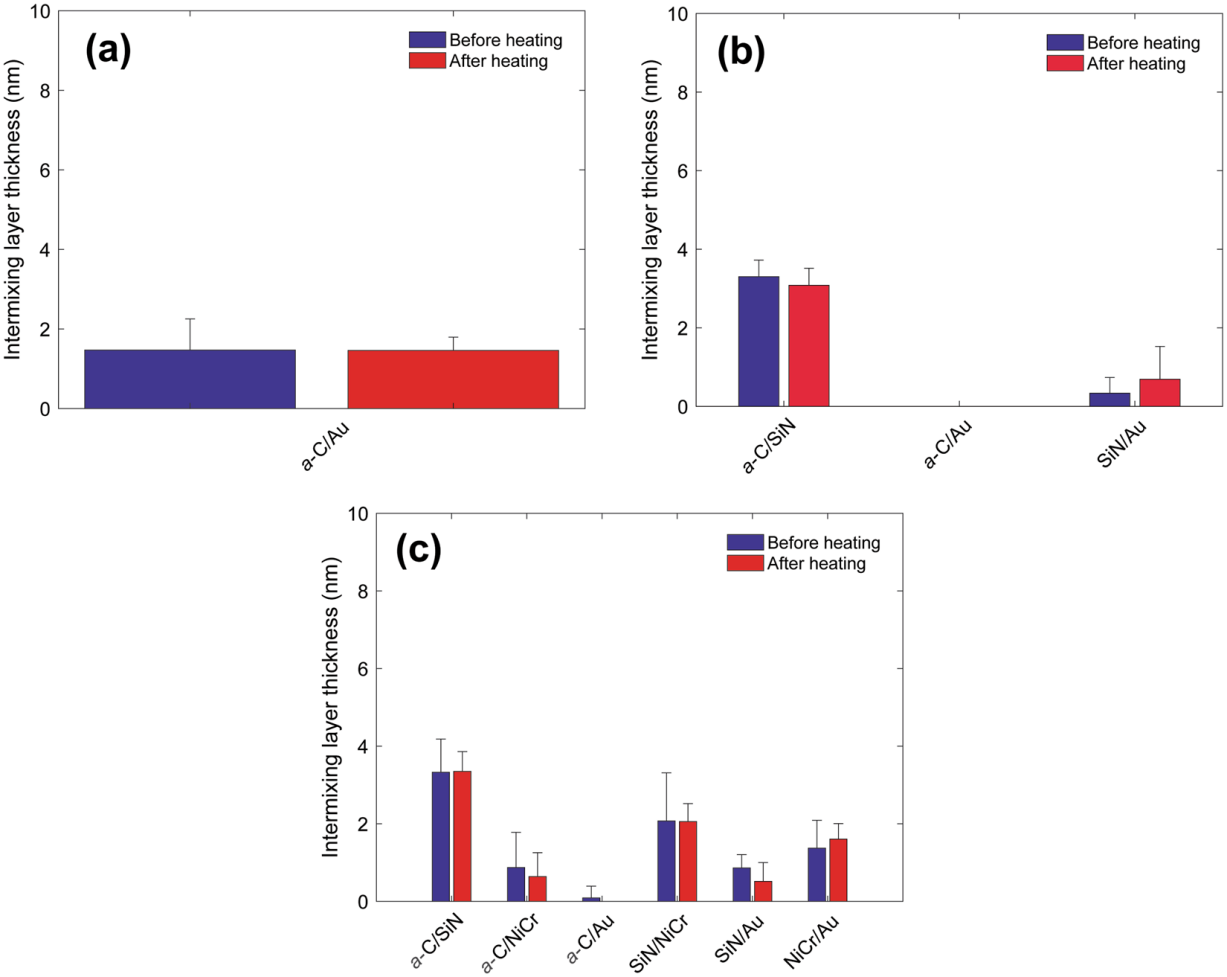
Intermixing layer thickness measured before and after heating at 350 °C for 30 min in an Ar environment for (**a**) industry sourced *a*-C overcoat deposited on a SiN/Au sample stack, (**b**) *a-*C overcoat deposited on a SiN/Au sample stack, and (**c**) *a-*C overcoat deposited on a SiN/NiCr/Au sample stack.

Figure 8 shows the thickness of intermixing layers in different stack configurations with an Au base layer before and after heating. No change in intermixing layer thickness was found for the industry sourced *a*-C overcoat in the *a*-C/SiN/Au stack sample after heating (Fig. 8a). The SiN layer in that sample stack could not be detected by EELS, likely indicating a negligible presence of this layer and/or discontinuous film deposition. This is supported by the formation of an intermixing layer between the *a*-C overcoat and the Au base layer. In contrast, insignificant intermixing was found between the *a*-C overcoat and the Au base layer (Fig. 8b,c). In fact, the most significant intermixing in the *a*-C/SiN/NiCr/Au stack configuration occurred between the *a*-C overcoat and the SiN adhesion layer, characteristic of the subplantation process^25^, which is intrinsic to deposition processes having energetic ions as film precursors, such as the FCVA method, and between the NiCr layer and the SiN and Au layers. Only minor changes in thickness of the intermixing layer in the latter stack configuration were observed after heating. These results suggest that the selected temperature and heating duration did not cause significant diffusion of elements between the layers and, more importantly, that the particular stack assembly was effective in confining C implantation and diffusion only in the SiN (predominantly) and NiCr (secondarily) layers.

**Figure 9:**
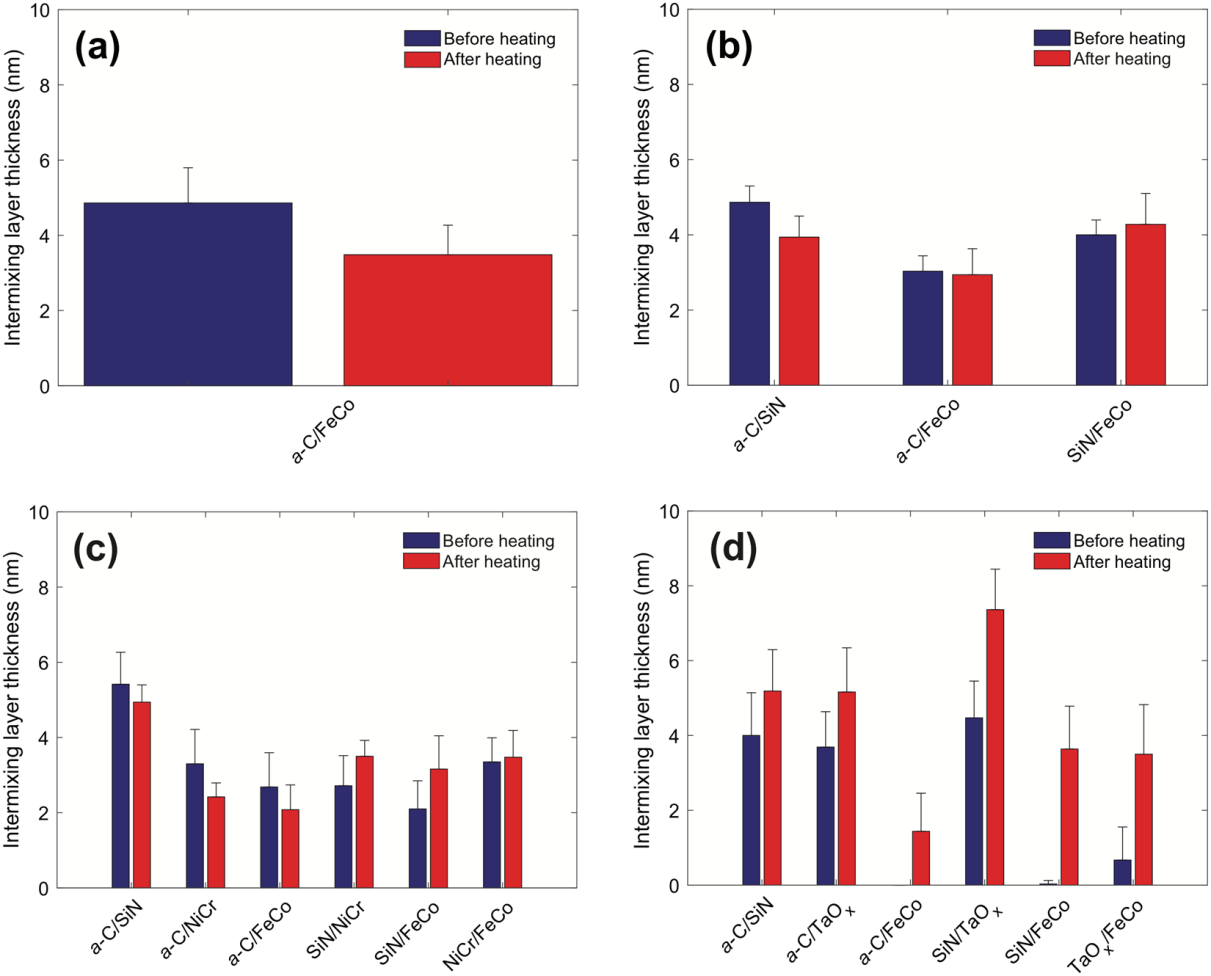
Intermixing layer thickness measured before and after heating at 350 °C for 30 min in an Ar environment for (**a**) industry sourced *a*-C overcoat deposited on a SiN/FeCo sample stack, (**b**) *a*-C overcoat deposited on a SiN/FeCo sample stack, (**c**) FCVA *a*-C overcoat deposited on a SiN/NiCr/FeCo sample stack, and (**d**) *a*-C overcoat deposited on a SiN/TaO_x_/FeCo sample stack.

Figure 9 shows the effect of heating on the thickness of intermixing layers in different stack configurations with a FeCo base layer. As for the Au base layer, the SiN layer was not effective in preventing intermixing of the *a*-C overcoat with the FeCo base layer (Fig. 9a,b), especially for the industry sourced stack in which the SiN layer was significantly thinner. However, unlike the Au base layer, heating caused an unexpected small decrease in intermixing between C and SiN in each sample (Fig. 9a–c), except for the sample having a TaO_x_ buffer layer (Fig. 9d). Moreover, intermixing of C with the FeCo base layer was either slightly reduced or suppressed in the presence of a NiCr (Fig. 9c) or TaO_x_ (Fig. 9d) buffer layer, respectively.

## Discussion

Carbon overcoats are generally sensitive to long-term heating, tending to graphitize, oxidize, and disintegrate at elevated temperatures^13,14^. Moreover, the protective capability of a carbon film strongly depends on its uniformity (continuity), structural instability (e.g., *sp*^3^→*sp*^2^ rehybridization, graphitization, and oxidation), and interdiffusion characteristics^12,16^. The intense laser heating encountered during steady-state HAMR read/write conditions may degrade the nanostructure and stability of the *a*-C overcoat through carbon diffusion into the underlying FeCo and Au layers. Another possibility is overcoat delamination due to high thermal stresses generated by the thermal expansion mismatch between the carbon overcoat and metal base material and the weak adhesion of carbon to a metal surface. Therefore, the structural stability, oxidation/interdiffusion resistance, and mechanical integrity of the protective carbon overcoat are critical challenges to the reliability of devices operating at elevated temperatures, such as HAMR heads. Motivated by the foregoing critical issues, the main objective of this study was to introduce a new class of layered coatings for HAMR heads, which have the potential to overcome the aforementioned limitations of single *a*-C overcoats. To enhance the adhesive strength of the overcoat and inhibit carbon interdiffusion into the underlying metal substrate and/or oxidation, a SiN adhesion layer^9^ and a NiCr or TaO_x_ buffer layer^10,11^ were deposited between the *a*-C overcoat and the Au or FeCo base material. Thus, novel layered coatings with different stack configurations were synthesized by depositing thermally stable *a*-C overcoats onto adhesion-enhancing/oxidation-resistant layer stacks.

Another unique aspect of this study is the use of HRTEM/STEM and EELS to evaluate the structural stability (hybridization), measure the true thickness of the *a*-C overcoat, study the intermixing of different layers due to energetic C^+^ ion bombardment (subplantation process), and identify interdiffusion of elements across layer interfaces due to heating. The quantitative information acquired by combining these techniques cannot be obtained with diffraction-based techniques because the *a*-C overcoat is amorphous. In addition, although X-ray photoelectron spectroscopy (XPS), X-ray reflectivity (XRR), and visible resonant Raman spectroscopy have been widely used to examine the structure of *a*-C overcoats, these techniques are limited by the spatial resolution and weak signal intensity. In particular, XPS uses the chemical shift between the *sp*^2^ and *sp*^3^ components that are fitted to the C 1*s* spectrum to obtain information about the material structure, yielding an overall composition to a depth of ~10 nm. XRR cannot accurately measure the density of films with thickness less than ~20 nm. Raman is only sensitive to *sp*^2^ hybridization and is usually used to indirectly interpret the *sp*^3^/*sp*^2^ ratio in terms of the D-to-G band intensity ratio, which lacks accuracy especially when used for ultrathin films. Because of the high resolution of EELS (the binding energy gap between *sp*^2^ and *sp*^3^ is ~0.8 eV), this technique is suitable for examining the nanostructure of individual layers and detecting interdiffusion of C, Ni, Fe, or Au with an in-plane resolution of ~0.2 nm. Contrary to traditional film thickness measurement techniques relying on contrast differences, EELS yields the true film thickness and the thickness of intermixing regions, because it uses the elemental peaks in the high energy loss range to determine the boundaries of each layer. Thus, a comprehensive study of the thickness, nanostructure, intermixing, and interdiffusion of these novel layered coatings was performed by combining HRTEM/STEM with high-energy resolution EELS.

The cross-sectional HRTEM images and EELS depth profiles of different sample stacks obtained before and after heating (Figs 1–7) showed no changes in uniformity, minor variation in thickness, and a small decrease in *sp*^3^ fraction (Table 3) of the *a*-C overcoat due to heating, attributed to partial *sp*^3^→*sp*^2^ rehybridization. Moreover, the effect of heating on the thickness of the adhesion and buffer layers was found to be within the experimental error (Table 4), suggesting that element diffusion between these layers was secondary under the present heating conditions. The slightly thicker intermixing layer at the bottom of the *a*-C overcoats developed in this study compared with industry sourced *a*-C overcoats (Figs 8 and 9) is attributed to the subplantation process^25^, which plays a dominant role in film growth processes involving energetic ion bombardment. The thin SiN layer was not effective in preventing intermixing of C with the Au base layer during deposition (Fig. 8a) as opposed to the thicker SiN layer (Fig. 8b) and SiN/NiCr bilayer (Fig. 8c), which hindered C subplantation in the Au base layer. Only minor changes in the intermixing layer thickness occurred after heating, implying insignificant interdiffusion in all stack assemblies having an Au base layer. However, using the low energy loss peaks to identify Au intermixing only made this determination approximate. For the layered coatings deposited on a FeCo base layer, the SiN layer was relatively less effective in preventing intermixing of C with the base layer, especially the thinner SiN layer (Fig. 9a); however, in contrast to the coating assemblies with an Au base layer, heating caused thinning of the intermixing layer between C and SiN in all coatings (Fig. 9b,c), except for the sample having a TaO_x_ buffer layer (Fig. 9d), while intermixing of C with the FeCo base layer was either reduced or hindered in the presence of a NiCr (Fig. 9c) or TaO_x_ (Fig. 9d) buffer layer, respectively. Further studies are needed to fully understand the underlying reasons for these different trends in nanoscale interdiffusion between these ultrathin layers.

This study was focused on the structural stability and diffusion characteristics of layered coatings exposed to heating conditions determined from a conservative estimate of the temperature reached by the WP during extensive writing. Because the critical graphitization temperature of *a*-C films with 40% *sp*^3^ is ~400 °C^16^ and the *sp*^3^ content of most *a*-C overcoats examined in this study is ≥40% (Table 3), it may be inferred that the upper limit of the heating temperature of the present *a*-C overcoats should be >400 °C, which is outside the predicted temperature rise range of 100–300 °C in the vicinities of the NFT and WP elements. Moreover, all overcoats were deposited under optimal FCVA conditions, which have been proven to produce a bulk layer with relatively high *sp*^3^ content^17^. The dominance of the *sp*^3^-rich bulk layer over the *sp*^2^-rich intermixing and surface layers is critical to the overcoat’s thermal stability, oxidation, and wear resistance. However, decreasing the overcoat thickness below ~2 nm leads to a disproportional decrease of the bulk layer thickness relative to the intermixing and surface layers^17^, thereby dramatically depleting the overcoat’s protective capability. Consequently, further innovations in FCVA film deposition are needed to enable the growth of <2-nm-thick *a*-C overcoats with the desirable properties.

Although the *a*-C overcoats were found to be relatively stable in inert environments under HAMR operating temperatures, it is imperative to investigate the effects of oxygen on the stability of the overcoat and underlying adhesion and buffer layers. The presence of oxygen, even at low concentrations, can be problematic because the high air-bearing pressure generated during the operation of the drive may significantly increase the oxygen concentration at the head/disk interface, which in conjunction with the elevated operating temperature may accelerate dissociation and oxidation of the overcoat and the WP and hydrolyze the exposed NFT. Future studies should focus on the combined effects of elevated temperature and oxygen concentration on the oxidation/corrosion and diffusion resistance of layered overcoats with similar stack configurations.

## Conclusion

Repetitive thermal loading and interdiffusion across the *a*-C/NFT and *a*-C/WP interfaces of HAMR heads eventually destabilizes the protective *a*-C overcoat, causing corrosion of the WP and hydrolyzation of the NFT. To enable new breakthroughs in high-density data storage by the emerging HAMR technology, it is imperative to resolve all critical issues concerning the thermal stability and corrosion/diffusion resistance of the protective overcoat on the NFT and WP elements of HAMR heads. The present study represents a concerted effort to provide a solution to this problem by introducing novel ultrathin-layer stacks consisting of the overcoat, adhesion (seed) layer, and buffer (diffusion barrier) layer. The thermal (structural) stability of the *a*-C overcoat and intermixing/interdiffusion with the adhesion, buffer, and base (substrate) layers under heating conditions that mimic steady-state operation conditions of a HAMR head were examined by combining state-of-the-art microanalysis methods (HRTEM, STEM, and EELS). The results of various layered coating configurations were contrasted to assess the potential of this new class of layered coatings to protect HAMR heads against thermal damage.

The findings of this study show that heating of layered coatings consisting of *a*-C overcoat, SiN adhesion layer, NiCr or TaO_x_ buffer layer, and Au or FeCo base layer at 350 °C for 30 min in an inert Ar environment does not result in significant structural changes and thinning of the overcoat or excessive intermixing in most stacking configurations. The EELS measurements reveal slight *sp*^3^→*sp*^2^ rehybridization of the *a*-C overcoats after heating and minute changes in the thickness of intermixing layers, which is attributed to limited diffusion. Further investigations should explore the development of thinner *a*-C overcoats with increased *sp*^3^ contents to reduce rehybridization under typical HAMR operation temperatures and layer thickness optimization of such layered coatings in the presence of oxygen. In this context, it is important to consider the formation of an overcoat with a sufficiently thick *sp*^3^-rich bulk layer, which can preserve or even enhance the thermal stability of the *a*-C overcoat, and much thinner *sp*^2^-rich intermixing and surface layers to further reduce the overcoat thickness, in conjunction with the development of effective anti-diffusion ultrathin underlayers.

## Methods

### Deposition of base, buffer, and adhesion layers

Base layers consisting of Au (30 nm) or Fe-25%Co (80 nm) deposited on Si(100) wafers were coated with various adhesion and buffer layers. Three different layers, i.e., SiN, NiCr, and TaO_x_, were deposited onto the base layers by ion beam deposition. Specifically, a SiN (type I) adhesion layer was formed under Ar/N_2_ gas flow conditions using a Si_3_N_4_ target, a SiN (type II) adhesion layer was formed under Ar gas flow conditions using a Si_3_N_4_ target, a NiCr buffer layer was formed under Ar gas flow conditions using a Ni-20%Cr metal target, and a TaO_x_ buffer layer was formed under Ar/O_2_ gas flow conditions using a Ta metal target. Table 1 gives the layer composition and nominal thickness (measured from HRTEM images) for each stack assembly.

### Deposition of carbon overcoat

Before depositing the *a*-C overcoat in a custom-made FCVA system^23,26,27^, each sample was partitioned (~5 × 5 mm^2^) and rinsed in isopropanol for 10 min and then in acetone for another 10 min. *a*-C overcoat deposition included the following three main steps: (1) the vacuum chamber was pumped down to a low base pressure (<5 × 10^−7^ Torr) to remove any residual gases adsorbed onto the chamber walls, (2) the base pressure was reduced to <5 × 10^−7^ Torr, and (3) plasma arcing was induced at the cathode (99.99% pure graphite) surface by a mechanical striker. The plasma was stabilized by applying a cusp-configuration magnetic field to the cathode^26^. Out-of-plane S-shaped electromagnetic coils generated a magnetic field that prevented the deposition of macroparticles and/or droplets ejected from the cathode during plasma arcing. The current in the auxiliary, upstream, and downstream coils was set at ~32 A. Under these current-plasma conditions, only high-purity (~99.99%) C^+^ ions arrived at the filter exit. All of the *a*-C film depositions were performed under previously optimized FCVA conditions^17,28^, i.e., −75 V substrate bias voltage, 65% duty cycle of substrate pulse biasing, 1.48 × 10^19^ ions/m^2^·s ion flux, 10° ion incidence angle (measured from the surface normal), and 6 s deposition time (8.88 × 10^19^ ions/m^2^ ion dose). To enhance the overcoat uniformity, in all coating depositions the substrate holder was rotated at 60 rev/min. Finally, each sample was sequentially rinsed in isopropanol and acetone for 10 min to remove the adhesive residue underneath the Si(100) substrate.

### Sample heating

**Figure 10:**
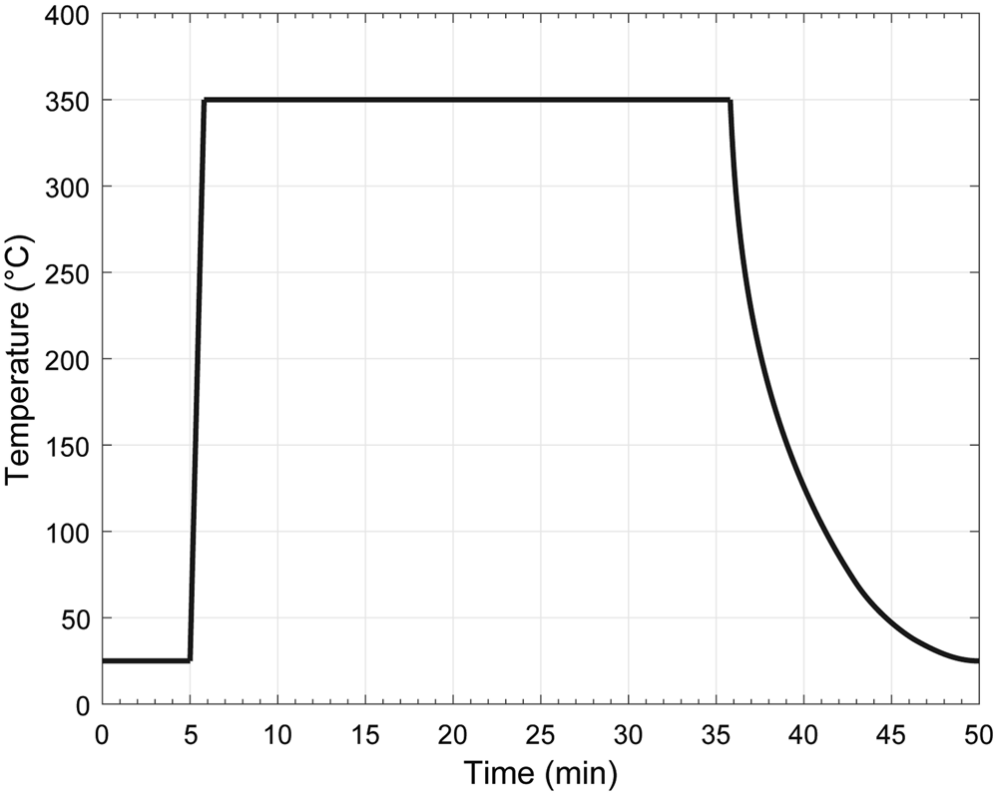
Temperature profile used to heat the samples at 350 °C in an environment of flowing Ar gas. After purging the chamber for 5 min with Ar gas, the temperature was ramped to 350 °C within 1 min and held constant for 30 min before turning off the heater and allowing the sample to cool down to room temperature for ~60 min before removing it from the chamber.

Following *a*-C overcoat deposition, the samples were placed in the chamber of a thermal processor (AW610, AccuThermo) and heated at 350 °C for 30 min under controlled Ar gas flow. Figure 10 shows the heating curve used in the treatment experiments. These heating conditions were selected based on a conservative estimate of the maximum temperature reached by the WP during continuous writing of a HAMR head. The samples were allowed to convectively cool down to <50 °C for ~60 min under the continuous flow of Ar gas before removing them from the chamber.

### Deposition of capping layer

A thin (20 nm) Cr capping layer was deposited on each *a*-C-coated sample in a radio-frequency (RF) sputtering system (Randex-2400, Perkin-Elmer) to facilitate the distinction of the *a*-C overcoat from the epoxy glue used to prepare the cross-sectional HRTEM samples. The deposition of the Cr capping layer comprised the following four main steps: (1) the vacuum chamber was pumped down to a low base pressure (<5 × 10^−6^ Torr) to remove any residual gases adsorbed onto the chamber walls, (2) Ar gas was introduced into the chamber at a flow rate of 61 sccm and a working pressure of 3 × 10^−3^ Torr was established, (3) the Cr target was cleaned by a 10-min Ar^+^ ion bombardment performed under RF sputtering conditions of 3 × 10^−3^ Torr working pressure, 250 W forward RF power, and 61 sccm Ar gas flow rate, and (4) the Cr layer was deposited onto the sample surface by sputtering the Cr target with Ar^+^ ions for 1 min.

### Microanalysis methods

Cross-sectional HRTEM samples were prepared by mechanical grinding, dimpling, and surface finishing using ion milling. The samples were glued face-to-face with M-bond 610 epoxy and cured at 160 °C for 90 min. Additional Si(100) wafer was epoxy-glued to both sides of the sandwiched samples to increase the total thickness to >3 mm and the epoxy was cured at 160 °C for 90 min. Then, using a diamond blade and a saw, the samples were sectioned into ~700- to 1000-μm-thick slices and cored to 3-mm-diameter disks. Subsequently, the disks were polished down on side to ~300 μm thickness by successively using smaller grits (from 30 to 0.1 μm) on a polishing system (MultiPrep, Allied High Tech). Finally, the center thickness was thinned down to <20 μm by a dimpler, and the disk was ion milled from both top and bottom sides with Ar^+^ ion guns (PIPS II, Gatan) operated at 4.5 kV. An Ar^+^ ion incidence angle of 5° was used to produce a through-thickness hole across the sample/epoxy/sample stack configuration.

HRTEM images and EELS spectra were obtained with a microscope (F20 UT, FEI Tecnai) operated at 200 kV, which was equipped with a CCD camera (2048 × 2048 pixels) positioned 42 mm behind a Gatan imaging filter. A 9.3-mrad C2 semi-angle, a 150-μm C2 aperture, and a 16.3-mrad EELS collection semi-angle were used in this study. Using the full width at half maximum of the zero-loss peak, the energy resolution was found to be in the range of 0.5–0.6 eV, which is sufficiently low for distinguishing the *sp*^2^ and *sp*^3^ hybridizations, given the bandgap difference between *sp*^2^ and *sp*^3^ is ~0.8–0.9 eV. The spatial resolution in the scanning TEM (without a monochromator) used in this study was 0.2 nm. For each sample, a scanning line was set perpendicular to the *a*-C overcoat and data for each EELS spectrum were collected over an area of 0.2 nm diameter. For carbon bonding analysis, the dispersion was set to 0.1, whereas the dwell time was varied between 1 and 2 s. For composition analysis, the dispersion was set to 0.5, while the dwell time was varied between 0.1 and 2 s (as needed) in order to capture the energy peak of elements of interest with minimal background noise. Each scan started in the Au or FeCo base layer and ended in the Cr capping layer.

### Calculation of hybridization fraction

The cross-section nanostructure (hybridization) and elemental composition of the *a*-C overcoats were determined from EELS measurements. EELS uses the inelastic electron-electron collisions between electron beam electrons and inner (core-shell) sample electrons that produce ionization edges to detect the presence of various elements. The C ionization edge is at 284 eV and reveals both *sp*^2^ and *sp*^3^ hybridization modes, which are identified by π^*^ and σ^*^ peaks in the energy range of 285–305 eV. The *sp*^2^ and *sp*^3^ fractions for a given sample were determined from the π^*^/σ^*^ ratio of the sample and the π*/σ* ratio of a standard sample (pure graphite), following the method used in a previous study^17^.

## Electronic supplementary material

Supplementary Information
