# Supplementary material for: Nanostructure, structural stability, and diffusion characteristics of layered coatings for heat-assisted magnetic recording head media
Source: Sci Rep. 2018 Jun 28;8:9807. doi: 10.1038/s41598-018-27688-4 (PMC6023885; doi:10.1038/s41598-018-27688-4)
Supplement: Supplementary file 1 — Supplementary Information [file 41598_2018_27688_MOESM1_ESM.pdf]

Nanostructure, structural stability, and diffusion characteristics of layered coatings for heat-assisted magnetic recording head media

J. Matlak,[1] E. Rismaniyazdi,[2] and K. Komvopoulos[1,*]
[1]*Department of Mechanical Engineering, University of California, Berkeley, CA 94720, USA*
[2]*Western Digital Company, San Jose, CA 95119, USA*

Supplementary Information

The EELS major edges, shown in Fig. S1, were used to identify the presence of various layers in the layered coatings. The peak intensity varies with the amount of material present.

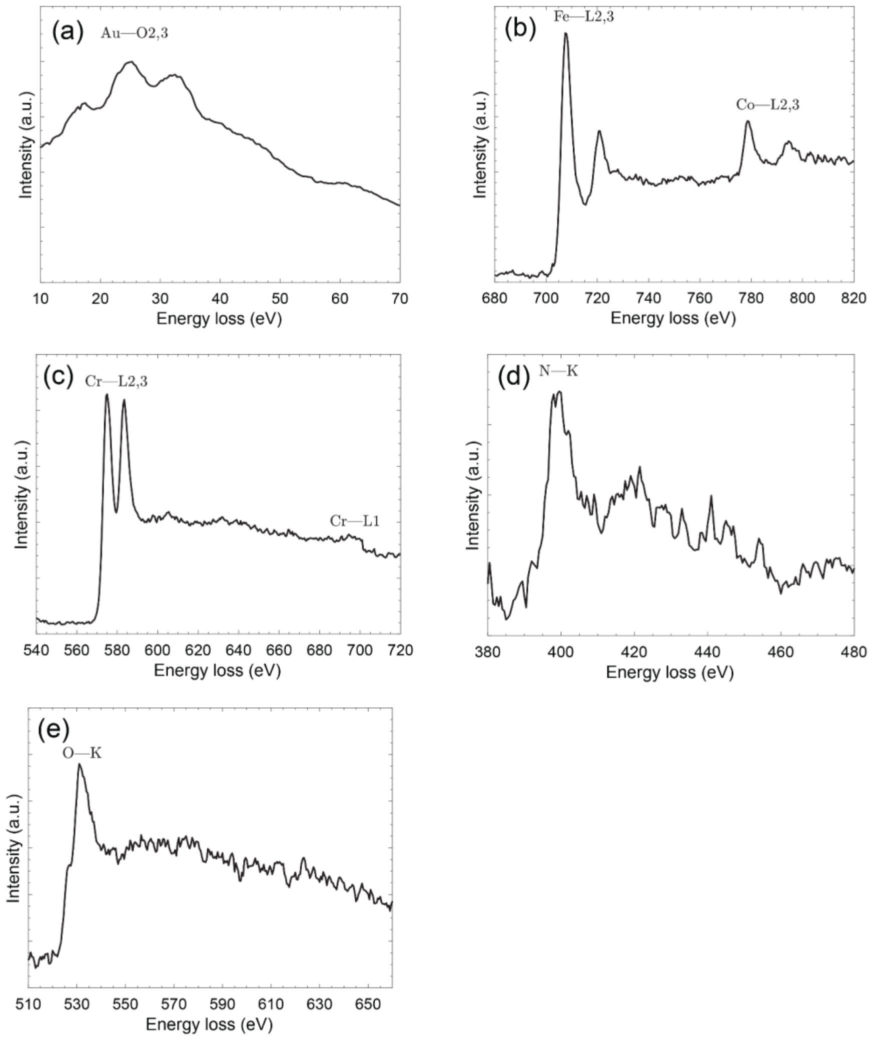

Fig. S1. EELS major edges used to identify the presence of various layers in the layered coatings: (a) Au, (b) FeCo, (c) NiCr, (d) SiN, and (e) TaO_x. (Source: Gatan, EELS Atlas, http://www.eels.info/atlas. Accessed Nov. 2017).

*Corresponding author: Tel. 510-642-2563; Fax: 510-642-5539; E-mail: kyriakos@me.berkeley.edu
